# COVID-19 trends at the University of Tennessee: predictive insights from raw sewage SARS-CoV-2 detection and evaluation and PMMoV as an indicator for human waste

**DOI:** 10.3389/fmicb.2024.1379194

**Published:** 2024-03-28

**Authors:** Ye Li, Kurt Ash, Isablla Alamilla, Dominique Joyner, Daniel Edward Williams, Peter J. McKay, Brianna Green, Sydney DeBlander, Carman North, Fadime Kara-Murdoch, Cynthia Swift, Terry C. Hazen

**Affiliations:** ^1^Department of Civil and Environmental Sciences, University of Tennessee, Knoxville, TN, United States; ^2^Biosciences Division, Oak Ridge National Laboratory, Oak Ridge, TN, United States; ^3^Battelle Memorial Institute, Columbus, OH, United States; ^4^Center for Environmental Biotechnology, University of Tennessee, Knoxville, TN, United States; ^5^Department of Microbiology, University of Tennessee, Knoxville, TN, United States; ^6^College of Natural Science, Michigan State University, East Lansing, MI, United States; ^7^Student Health Center, University of Tennessee, Knoxville, TN, United States; ^8^Department of Earth and Planetary Sciences, University of Tennessee, Knoxville, TN, United States; ^9^Institute for a Secure and Sustainable Environment, University of Tennessee, Knoxville, TN, United States

**Keywords:** SARS-CoV-2, PMMoV, COVID-19, raw wastewater, sewage, WBE, RT-qPCR

## Abstract

Wastewater-based epidemiology (WBE) has become a valuable tool for monitoring the prevalence of SARS-CoV-2 on university campuses. However, concerns about effectiveness of raw sewage as a COVID-19 early warning system still exist, and it’s not clear how useful normalization by simultaneous comparison of Pepper Mild Mottle Virus (PMMoV) is in addressing variations resulting from fecal discharge dilution. This study aims to contribute insights into these aspects by conducting an academic-year field trial at the student residences on the University of Tennessee, Knoxville campus, raw sewage. This was done to investigate the correlations between SARS-CoV-2 RNA load, both with and without PMMoV normalization, and various parameters, including active COVID-19 cases, self-isolations, and their combination among all student residents. Significant positive correlations between SARS-CoV-2 RNA load a week prior, during the monitoring week, and the subsequent week with active cases. Despite these correlations, normalization by PMMoV does not enhance these associations. These findings suggest the potential utility of SARS-CoV-2 RNA load as an early warning indicator and provide valuable insights into the application and limitations of WBE for COVID-19 surveillance specifically within the context of raw sewage on university campuses.

## Introduction

Wastewater-Based Epidemiology (WBE) emerges as a valuable tool for the early detection and surveillance of COVID-19 outbreaks within university communities. As of December 22, 2023, a study by researchers at the University of California, Merced, reveals that 289 universities across 72 countries, encompassing 4,648 sites, have adopted WBE practices (WBE Collaborative, 2023). Through routine analysis of wastewater samples from dormitories and other campus facilities, health authorities can promptly identify potential infection clusters and implement preventive measures. This proactive approach curtails virus transmission among students, facilitating swift isolation and contact tracing, ultimately mitigating the impact of outbreaks on campus life.

The significant early warning capability of WBE is evident in COVID-19 surveillance, encompassing two crucial dimensions. Firstly, it signals the onset of an outbreak at its initial stage, and secondly, it forecasts an impending surge in the number of infected individuals. Noteworthy studies highlight the effectiveness of WBE in providing advanced indications of COVID-19 outbreaks (Rotondo et al., 2022; Contini et al., 2023), with reported early warning days varying from two days to three weeks for early COVID-19 trend detection. Karthikeyan et al. (2021) demonstrated accurate predictions of cases by a week and intermediate accuracy at three weeks using city wastewater. Similarly, Randazzo et al. (2020) identified positive SARS-CoV-2 results in wastewater samples twelve to sixteen days before the official reporting of COVID-19 cases in three out of seven wastewater catchment regions. Medema et al. (2020) identified a similar pattern, with wastewater samples indicating the presence of SARS-CoV-2 six days before the official reporting of the first cases. This detection was explicitly associated with the N3 gene, excluding the N1, N2, and E genes. Nemudryi et al. (2020) found SARS-CoV-2 RNA levels in wastewater exhibiting a lead time of two to four days before clinical results, and they were indicative of symptom onset by five to eight days. Ahmed et al. (2020a) emphasized the potential of wastewater monitoring as an early warning system, providing insights into the broader circulation of SARS-CoV-2, particularly in individuals with mild or no symptoms. However, Gerrity et al. (2021) propose that while wastewater surveillance may effectively function as a leading indicator for COVID-19 outbreaks, it may lag as an indicator for declining infection rates due to prolonged viral shedding. This introduces uncertainty concerning the timing and dynamics of infection rate trends as reflected in wastewater data. Moreover, most of the studies primarily examined wastewater rather than raw sewage when making early warning predictions. Additionally, Li et al. (2021) summarized that various methodological challenges can affect the accuracy of prevalence estimation in WBE.

One of the primary challenges in handling SARS-CoV-2 data revolves around the crucial normalization process. Raw sewage, which comprises a diverse range of liquids originating from toilets, sinks, showers, and dishwashing within a building, forms a complex matrix containing human waste components such as feces, urine, sputum, and nasal discharge. The viral levels present in sewage exhibit variability dependent on water usage patterns. To comprehensively understand such sewage, measurements of indicators associated with human waste or fluids are conducted simultaneously with the assessment of SARS-CoV-2 RNA load. Pepper Mild Mottle Virus (PMMoV) is systematically assessed with wastewater due to its acknowledged stability and minimal RNA load variation over time (Li et al., 2023a,b). This virus has been employed to detect pathogenic enteric viruses, as heightened RNA load of PMMoV often indicate increased levels of fecal contamination (Kitajima et al., 2018). Several studies consistently indicate that normalizing SARS-CoV-2 RNA load by PMMoV enhances correlations with COVID-19 cases at the community level. For example, Maal-Bared et al. (2023) observed that normalization led to an increase in *r_s_* by PMMoV in two wastewater treatment plants (WWTPs). Jafferali et al. (2021) recognized PMMoV as functionally effective, deeming it a potentially suitable internal reference standard for normalizing SARS-CoV-2 RNA load. Zhan et al. (2022) also reported improved correlations with COVID-19 cases when normalizing SARS-CoV-2 levels by PMMoV in campus data. However, an increasing number of reports cast doubt on the assumption that normalization by PMMoV contributes to the improvement of standardization and reporting in WBE data. Ai et al. (2021) reported that the normalization of the SARS-CoV-2 RNA load by PMMoV did not improve the correlation. Feng et al. (2021) reported that SARS-CoV-2 RNA load normalized by PMMoV reduced correlations in 8 out of 12 WWTPs. Nagarkar et al. (2022) also observed that the utility of PMMoV to normalize SARS-CoV-2 RNA load is not universal. These findings highlight the ongoing debate and complexity surrounding using PMMoV as a normalization factor in interpreting SARS-CoV-2 data in WBE. Additionally, although the associations between SARS-CoV-2 RNA load and COVID-19 cases are increasingly reported in the literature, it is noteworthy that most studies primarily concentrate on wastewater treatment plants (WWTPs).

This study aims to assess and compare the effects of PMMoV normalization on the correlations and early warning abilities related to SARS-CoV-2 RNA load in raw sewage and various factors, such as active COVID-19 cases, self-isolations, and their combination among all resident students at the University of Tennessee, Knoxville, over the course of one academic year. The findings of this study could enhance our comprehension of the potential applications and limitations of wastewater monitoring for COVID-19 surveillance, particularly within the context of raw sewage, on university campuses.

## Materials and methods

### Raw sewage sampling

In the summer of 2020, the University of Tennessee adopted WBE to proactively detect COVID-19 cases in student residence halls. These halls housed 7,486 individuals in Fall 2020 and 6,781 in Spring 2021, representing a microcosm of the campus community, which consisted of 30,559 students during the 2020 academic year. Over the course of the academic year from September 14, 2020, to September 21, 2021, sewage samples were regularly collected from 18 dormitories, 15 fraternities, and 14 sororities. Detailed information regarding the occupants of these student residence halls during the fall 2020 and spring 2021 semesters can be found in Table 1. Samples were taken downstream of dispense valves or sewer manholes just before merging with other sewer lines to ensure targeted sampling from buildings with distinct student populations. Sampling commenced at 8:00 am, which corresponds to a time when human activity typically surges, potentially leading to increased viral shedding from individuals and subsequently higher viral loads in the collected samples (Li et al., 2023a). Grab samples (>50 mL) were collected using a stainless-steel telescopic rod pole swivel dipper water swing sampler or a sterile Nalgene bottle. Samples were promptly transported to a Biosafety Level-2 laboratory within a cooler containing ice for processing within three hours of collection. Data utilized for analysis spanned from November 9, 2020, to June 4, 2021, coinciding with the commencement of detection for PMMoV.

**Table 1:** Characteristics of student residence halls in the University of Tennessee, Knoxville.

Building type	Average of age in years	Building	Sampling point	Occupancy^a^	
				20 Fall	21 Spring
Dorms	19.13	D1	Direct Dispense from valve	422	413
		D2	Direct Dispense from valve	371	340
		D3	Direct Dispense from valve	440	387
		D4	Direct Dispense from valve	458	330
		D5	Direct Dispense from valve	256	198
		D6	Direct Dispense from valve	256	196
		D7	Direct Dispense from valve	346	469
		D8	Direct Dispense from valve	386	19
		D9	Direct Dispense from valve	279	254
		D10	Direct Dispense from valve	547	529
		D11	Direct Dispense from valve	224	227
		D12	Direct Dispense from valve	224	227
		D13	Manhole	595	590
		D14	Direct Dispense from valve	10	10
		D15	Direct Dispense from valve	612	580
		D16	Direct Dispense from valve	10	10
		D17	Direct Dispense from valve	668	629
		D18	Direct Dispense from valve	535	576
Sum				6,639	5,984
Fraternities	20.33	F1	Manhole	11	0
		F2	Manhole	34	34
		F3	Manhole	25	23
		F4	Manhole	32	32
		F5	Manhole	11	11
		F6	Manhole	21	20
		F7	Manhole	27	27
		F8	Manhole	29	27
		F9	Manhole	18	18
		F10	Manhole	32	31
		F11	Direct Dispense from valve	10	14
		F12	Direct Dispense from valve	15	20
		F13	Manhole	11	11
		F14	Manhole	27	25
		F15	Manhole	16	14
Sum				319	307
Sororities	19.85	S1	Manhole	41	38
		S2	Direct Dispense from valve	47	40
		S3	Manhole	30	30
		S4	Manhole	42	42
		S5	Manhole	49	34
		S6	Manhole	35	35
		S7	Manhole	33	33
		S8	Manhole	32	36
		S9	Direct Dispense from valve	44	43
		S10	Manhole	50	37
		S11	Manhole	36	36
		S12	Direct Dispense from valve	31	30
		S13	Manhole	28	26
Sum				528	490

^a^Occupancy varied throughout the Fall 2020 and Spring 2021 semester, so the highest numbers of residents at any given time throughout the semester are shown.

### Sample processing

The initial RNA load of SARS-CoV-2 and PMMoV RNA was promptly determined from 50 mL of thoroughly mixed raw sewage samples within 5 h of collection. Detailed information regarding the sampling procedure is available in the work by Ash et al. (2023). Subsequently, the raw sewage samples underwent a pasteurization process for 2 h at 60°C, followed by centrifugation at 5,000 × *g* for 10 min and filtration through 0.45 μm and 0.22 μm nitrocellulose filters to eliminate large suspended particulate matter. The filtered samples were concentrated using ultrafiltration with an Amicon Ultra-15 filtration device (EMD Millipore, Burlington, MA). The Amicon Ultra was centrifuged at room temperature at 4,000 × *g* for 30 min (Swing-arm rotor) or 5,000 × *g* for 20 min (Fixed-angle rotor). The resulting concentrate, approximately (~250 μL), was transferred to 2 mL DNA LoBind tubes, and RNA extractions were performed using a Qiagen viral RNA Mini Kit (Qiagen, Valencia, CA, USA). In brief, 60 μL of RNA was extracted from a homogenized sample using the Qiagen viral RNA Mini Kit, following the manufacturer’s instructions. This process involved initial lysis of the sample containing viral RNA, followed by binding the RNA to a specialized spin column where contaminants were removed through washing steps. After drying the column, the purified RNA was eluted using an elution buffer, resulting in concentrated RNA suitable for RT-qPCR. DNase/RNase-free water served as the extraction negative control. Throughout the procedure, precautions were taken to minimize the potential for inhibitors that could interfere with downstream RT-qPCR analysis. All RNA samples were stored at −80°C and subjected to RT-qPCR analysis within one day of RNA extraction.

### RT-qPCR

**Figure 1:**
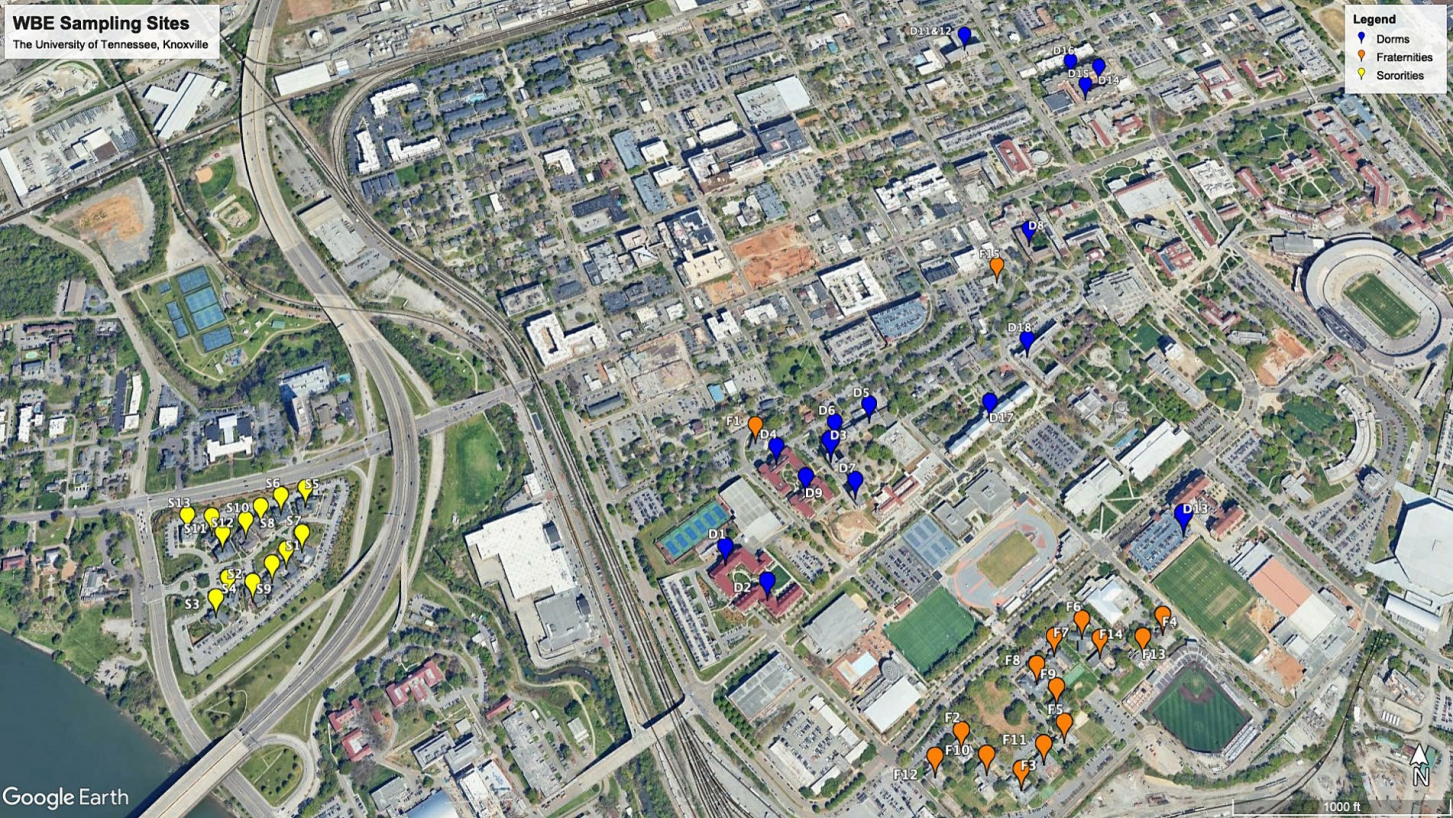
WEB sampling sites in the University of Tennessee, Knoxville.

RT-qPCR was employed to quantify the RNA load of SARS-CoV-2 and PMMoV RNA in each sample. Comprehensive details regarding the RT-qPCR methodology can be found in the publication by Ash et al. (2023). The CDC primer/probe assay for SARS-CoV-2 N1 was utilized, and quantification was conducted using the TaqPath 1-Step RT-qPCR Master Mix, CG (Thermo Fisher Scientific) on an Applied Biosystems QuantStudios 7 Pro Real-Time PCR System instrument (Figure 1).

Each 20 μL reaction comprised 5 μL of 4X Master Mix (Thermo Fisher Scientific), 0.25 μL of 10 μmol/L probe, 1 μL each of 10 μmol/L forward and reverse primers, 7.75 μL of nuclease-free water, and 5 μL of nucleic acid extract. The reagents were pipetted into 96-well plates and vortexed for 10 s. Thermocycling conditions were as follows: uracil-DNA glycosylase incubation for 2 min at 25°C, reverse transcription for 15 min at 50°C, activation of the Taq enzyme for 2 min at 95°C, and two-step cycling for 3 s at 95°C and 30 s at 55°C for 45 cycles. A positive test result was defined as an exponential fluorescent curve that crossed the threshold within 40 cycles (cycle threshold [Ct] <40).

PMMoV quantification was performed using RT-qPCR with the TaqPath 1-Step RT-qPCR Master Mix, CG (Thermo Fisher Scientific) on a QuantStudios 7 Pro instrument. In each 20 μL reaction, the components included 5 μL of 4X Master Mix (Thermo Fisher Scientific), 0.5 μL of 10 μmol/L probe, 1.8 μL each of 10 μmol/L forward and reverse primers, 8.9 μL of nuclease-free water, and 2 μL of nucleic acid extract. The reagents were pipetted into 96-well plates and vortexed for 10 s. Thermocycling conditions were as follows: uracil-DNA glycosylase incubation for 2 min at 25°C, reverse transcription for 15 min at 50°C, activation of the Taq enzyme for 10 min at 95°C, and two-step cycling for 30 s at 95°C and 1 min at 60°C for 40 cycles.

Each RT-qPCR run incorporated a series of three positive and negative controls, where the positive control comprised Mastermix and DNase/RNase-free water. All RT-qPCR reactions were conducted in triplicate, and results were considered valid only if the positive control yielded a positive outcome and the negative control remained negative. A sample was deemed positive only when all replicates were positive, each falling within the linear range of the standard curve.

The efficiency of the N1 standard curve was determined to be 94.7% (R^2^ = 1). The final quantification of SARS-CoV-2 RNA was calculated as the mean of three replicates of virus copies. The RT-qPCR outputs were subsequently converted to copies per liter. Notably, the detection limit for SARS-CoV-2 and PMMoV in this study was established at 20 and 10 copies/L, respectively (Bustin et al., 2009).

### Data analysis

Statistical analysis was conducted to investigate the relationship between SARS-CoV-2 RNA load and reported cases during a significant event. Data reflecting daily new cases at residence-specific levels were utilized for statistical analysis from November 9, 2020, to June 04, 2021. To facilitate the analysis, weekly case counts were aggregated in three ways: raw sewage sample collection one week before, during the week of, and one week after active cases and self-isolations. Spearman’s correlation assessed the relationship between raw and PMMoV-normalized SARS-CoV-2 RNA load and the aggregated case counts. The use of Spearman’s correlation avoided the assumptions of normality and the absence of outliers associated with the Pearson correlation (Hauke and Kossowski, 2011). The correlation coefficient’s magnitude indicated the association’s strength and direction, measuring how closely the points aligned along the monotonic association. Non-detectable values in viral data were considered as twenty for statistical purposes, aligning with the detection limitation of N1.

## Results

### Active and isolation clinical COVID-19 cases

**Figure 2:**
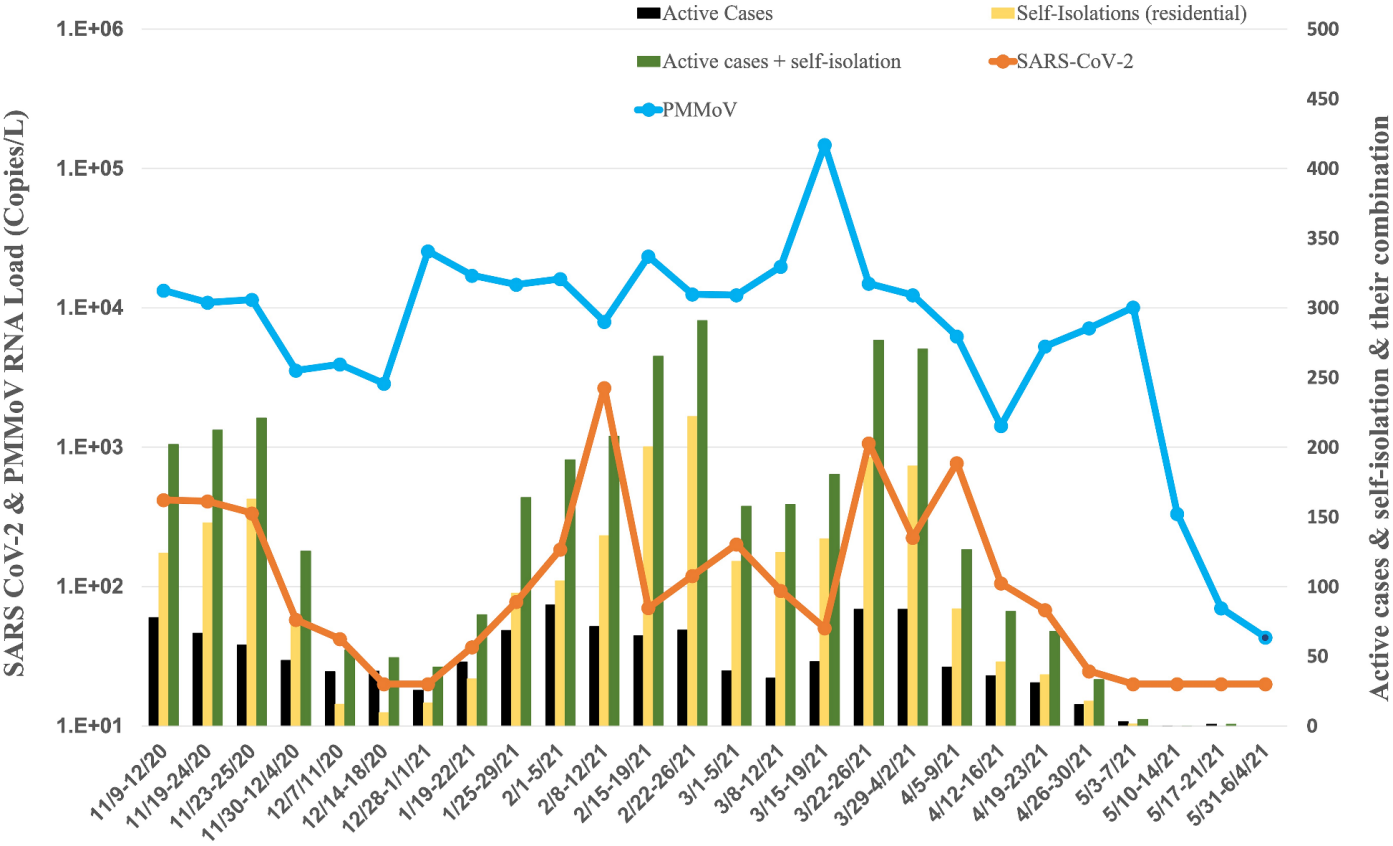
Average RNA load of SARS-CoV-2 in all residence halls, and active cases, and self-isolation students in the residential halls.

The Student Health Center provides valuable insights into the prevalence of COVID-19 on campus, reporting both active and isolated cases. Throughout the study period, an analysis of the data reveals a cumulative total of 2,321 active cases and 4,202 isolated cases spread across the 46 residence halls from September 14, 2020, to June 04, 2021 (Figure 2). The onset of the pandemic at the campus was marked by a significant surge in COVID-19 cases during the initial wave in September, with 778 active cases and 1,480 isolated cases. An in-depth analysis of specific timeframes throughout the study period reveals distinct waves of COVID-19 incidence. Following the September surge, subsequent months displayed varying degrees of activity. In October, there were 208 active cases and 345 isolation cases, indicating a notable decline from the initial peak. November saw a resurgence with 310 active cases and 628 isolation cases, suggesting a renewed increase in viral spread. December recorded 144 active cases and 55 isolation cases, signaling a potential decrease in cases toward the end of the year. As the new year began, January experienced a further rise with 170 active cases and 161 isolation cases. The trend continued in February with 292 active cases and 663 isolation cases, reaching another peak. March and April demonstrated a gradual decline, with 288 active cases, 757 isolation cases, 125 active cases, and 185 isolation cases, respectively. May saw a minimal presence, with only five active cases and two isolation cases, indicating a substantial reduction in COVID-19 incidence. Notably, during the winter break, spanning from December 12, 2020, to January 13, 2021, there remained a cumulative total of 160 active cases and 71 isolation cases, underscoring the persistent impact of the virus even during the holiday period.

### The RNA load of SARS-CoV-2 and PMMoV at different building types

**Figure 3:**
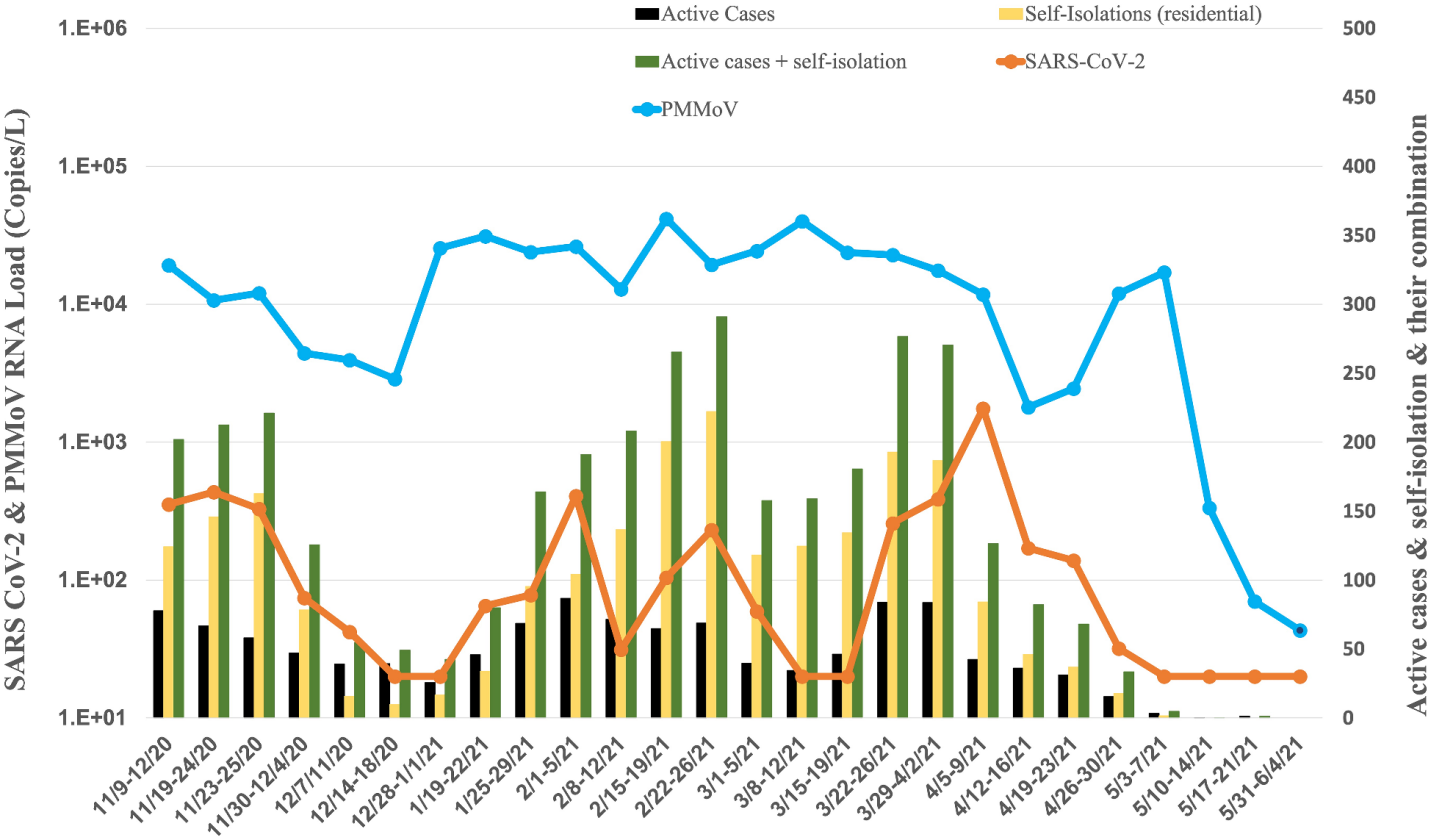
Average RNA load of SARS-CoV-2 in dorms, and active cases, and self-isolation student in the residential halls.

**Figure 4:**
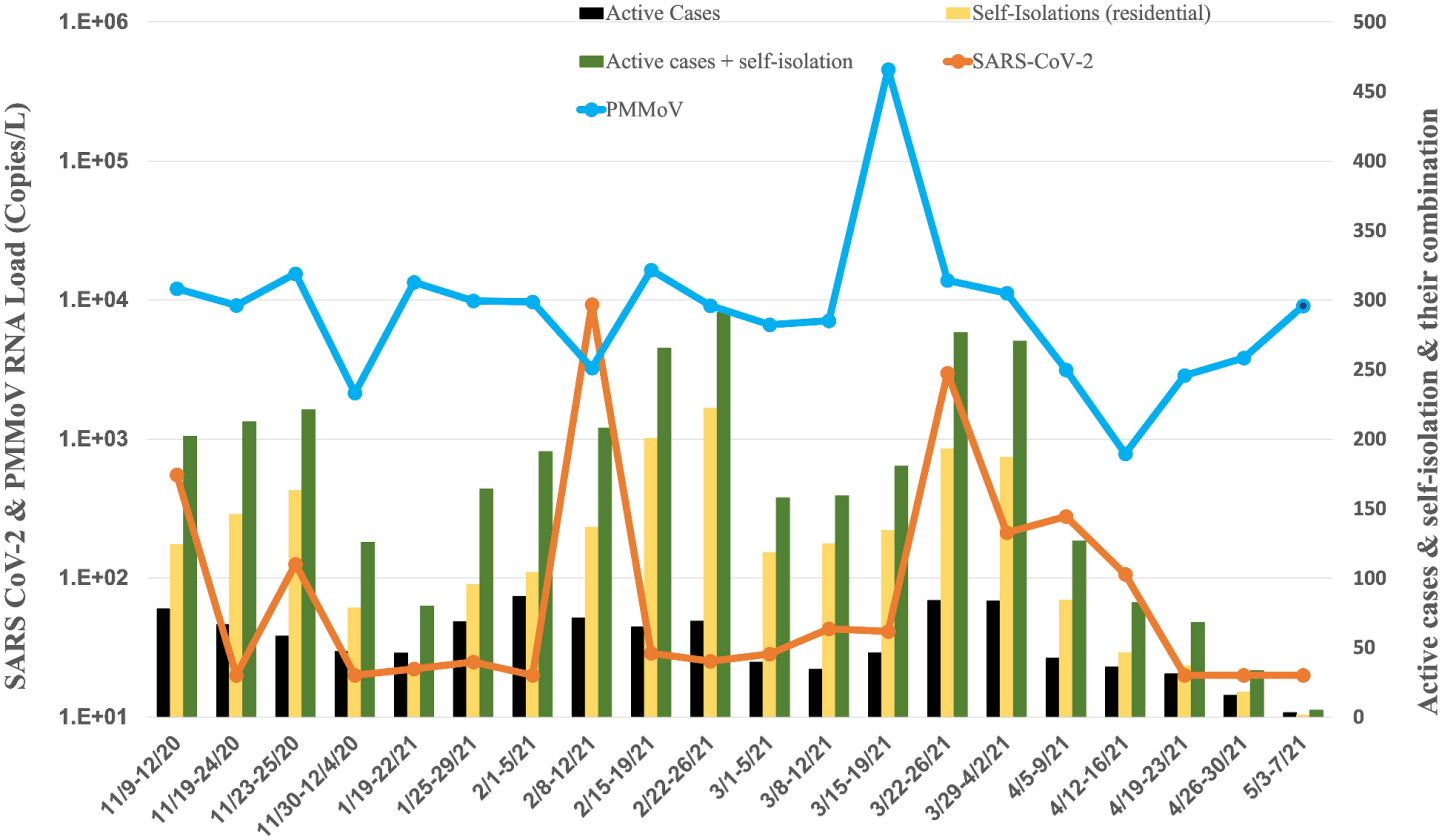
Average RNA load of SARS-CoV-2 in fraternity, and active cases, and self-isolation student in the residential halls.

**Figure 5:**
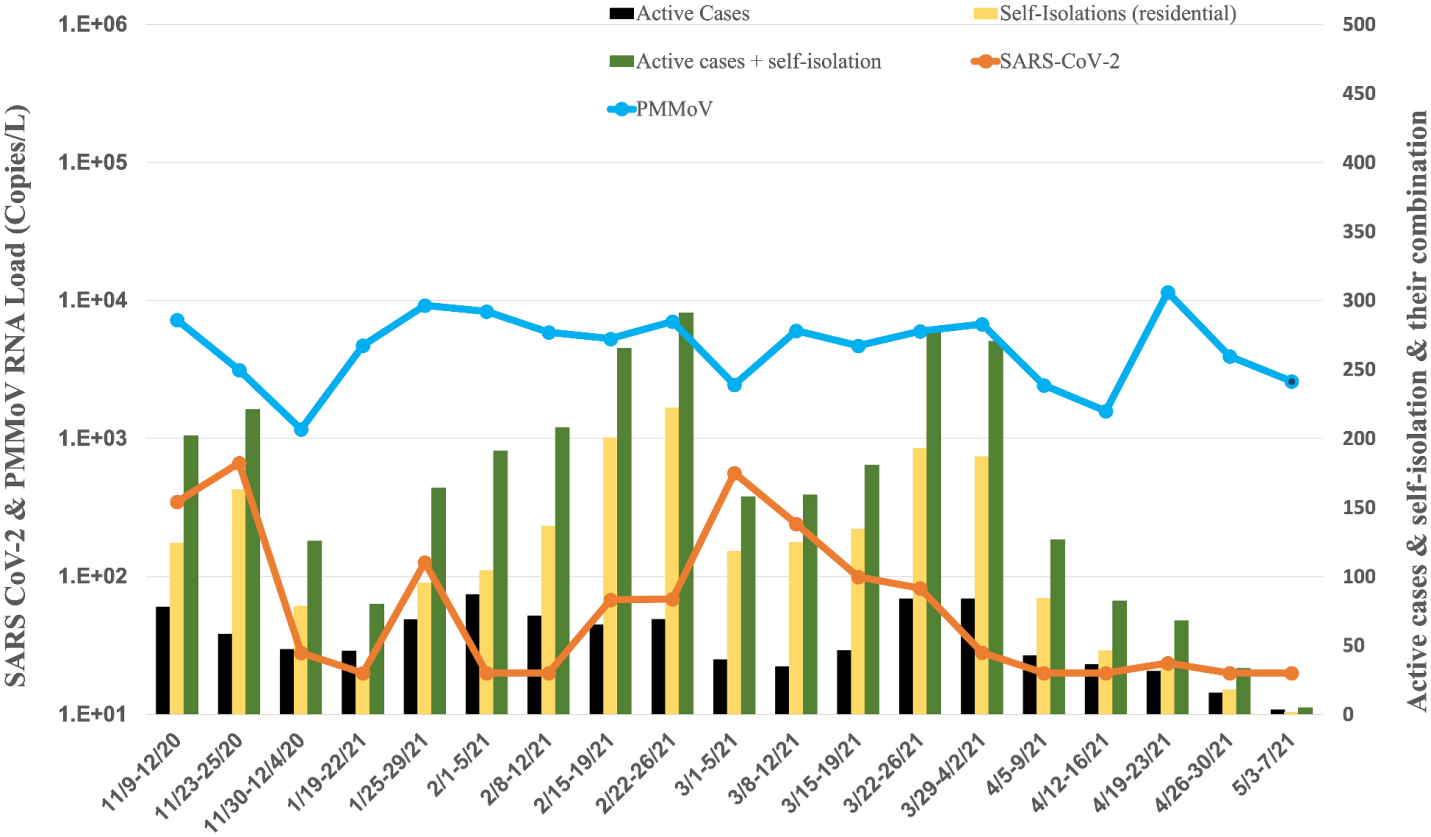
Average RNA load of SARS-CoV-2 in sororities, active cases, and self-isolation students in the residential halls.

The university implemented raw wastewater surveillance as a complementary strategy to clinical testing, aiming to detect the presence of SARS-CoV-2 on campus. This approach is particularly valuable in capturing individuals who may be asymptomatic or have mild symptoms and thus might not actively seek clinical screening. A comprehensive overview of the WBE project at the University of Tennessee, Knoxville, is provided in our paper by Ash et al. (2023). Figures 2–5 illustrate the measured viral copies per liter (copies/L) of the N1 genes across all residence halls, dormitories, fraternities, and sororities. The RNA load of SARS-CoV-2 RNA in raw sewage samples were 2.75 × 10^2^ ± 5.49 × 10^2^ copies/L for all residence halls, 1.97 × 10^2^ ± 3.48 × 10^2^ copies/L for dormitories, 6.93 × 10^2^ ± 2.12 × 10^3^ copies/L for fraternities, and 1.30 × 10^2^ ± 1.91 × 10^2^ copies/L for sororities. Correspondingly, the RNA load of PMMoV RNA across all samples were 1.55 × 10^4^ ± 2.79 × 10^4^, 1.57 × 10^4^ ± 1.20 × 10^4^, 3.07 × 10^4^ ± 9.98 × 10^4^, and 5.26 × 10^3^ ± 2.74 × 10^3^ copies /L for all residence halls, dormitories, fraternities, and sororities, respectively.

### All residence halls

Analyzing data of all residence halls (covering 18 dormitories, 15 fraternities, and 14 sororities) from November 2020 to May 2021, the temporal dynamics revealed fluctuating RNA load (Figure 2). Notably, in November 2020, the RNA load was 3.06 × 10^2^ ± 1.70 × 10^2^ copies/L, followed by a decrease in December 2020 to 2.74 × 10^1^ ± 1.28 × 10^1^ copies/L. January 2021 showed a slight increase to 5.74 × 10^1^ ± 2.90 × 10^1^ copies/L, while February 2021 witnessed a notable rise to 7.61 × 10^2^ ± 1.27 × 10^3^ copies/L. Subsequent months displayed varying RNA load, with March at 3.28 × 10^2^ ± 4.21× 10^2^ copies/L, April at 2.43 × 10^2^ ± 3.55 × 10^2^ copies/L, and May at non-detectable.

**Figure 6:**
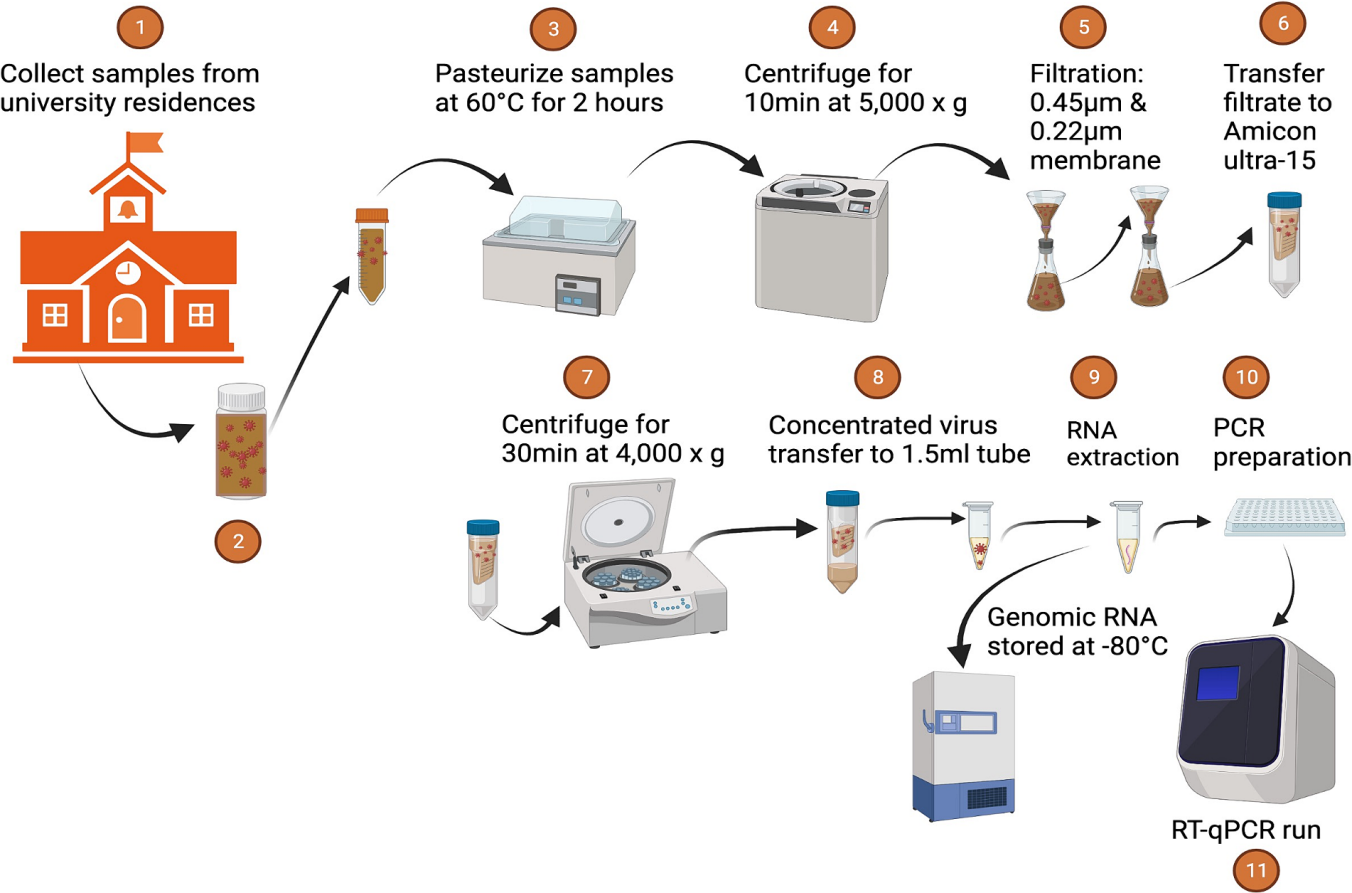
Sample processing.

The RNA load of PMMoV also exhibited variations across all residence halls during the same period (Figures 2–4, 6). In November 2020, the PMMoV RNA load was 9.84 × 10^3^ ± 4.32 × 10^3^ copies/L, increasing in December 2020 to 1.08 × 10^4^ ± 1.28 × 10^4^ copies/L. January 2021 recorded a further increase to 1.59 × 10^4^ ± 1.70 × 10^3^ copies/L. February 2021 exhibited a relatively stable RNA load at 1.50 × 10^4^ ± 6.53 × 10^3^ copies/L. March 2021 substantially increased to 4.15 × 10^4^ ± 5.97 × 104 copies/L. April 2021 decreased to 5.04 × 10^3^ ± 2.53 × 10^3^ copies/L, and in May 2021, the RNA load was 2.63 × 10^3^ ± 4.97 × 10^3^ copies/L.

### Dormitories

The RNA load of SARS-CoV-2 in 18 dormitories exhibited temporal variations from November 2020 to May 2021 (Figure 3). In November 2020, the RNA load were 2.98 × 10^2^ ± 1.56 × 10^2^ copies per liter (copies/L), indicating a moderate level. This was followed by a notable decrease in December 2020, with a RNA load of 2.74 × 10^1^ ± 1.28 × 10^1^ copies/L, suggesting a significant reduction. January 2021 showed a slight increase to 7.16 × 10^1^ ± 9.22 × 10^1^ copies/L, followed by a moderate rise in February 2021, with a RNA load of 1.94 × 10^2^ ± 1.65 × 10^3^ copies/L. March 2021 witnessed a similar level at 1.49 × 10^2^ ± 1.66× 10^2^ copies/L, while April 2021 displayed a substantial increase to 5.25 × 10^2^ ± 8.23 × 10^2^ copies/L. In May 2021, the RNA load decreased to a non-detectable level.

Concurrently, the RNA load of PMMoV in dormitories exhibited variations from November 2020 to May 2021 (Figure 3). In November 2020, the PMMoV RNA load was 1.16 × 10^4^ ± 6.08 × 10^3^ copies/L. December 2020 showed a similar level at 1.08 × 10^4^ ± 1.28 × 10^4^ copies/L. January 2021 increased to 2.75 × 10^4^ ± 5.10 × 10^3^ copies/L. February 2021 exhibited a relatively stable RNA load at 2.50 × 10^4^ ± 1.23 × 10^4^ copies/L. March 2021 displayed a similar level at 2.57 × 10^4^ ± 8.42 × 10^3^ copies/L. April 2021 decreased to 7.00 × 10^3^ ± 5.63 × 10^3^ copies/L, and in May 2021, the RNA load was 4.38 × 10^3^ ± 8.45 × 10^3^ copies/L.

### Fraternities

The RNA load of SARS-CoV-2 in 15 fraternities exhibited dynamic changes from November 2020 to May 2021 (Figure 4). In November 2020, the RNA load was 1.80 × 10^2^ ± 2.53× 10^2^ copies per liter (copies/L). January 2021 decreased to 2.36 × 10^1^ ± 1.89 × 10^0^ copies/L, indicating a substantial reduction. February 2021 recorded a significant increase to 2.34× 10^3^ ± 4.62 × 10^3^ copies/L, suggesting a resurgence. The RNA load remained elevated in March 2021 at 6.60 × 10^2^ ± 1.30 × 10^3^ copies/L. April 2021 displayed a moderate level at 11.06 × 10^2^ ± 1.21× 10^2^ copies/L; in May 2021, the RNA load was non-detectable.

Simultaneously, the RNA load of PMMoV in fraternities exhibited varying levels from November 2020 to May 2021 (Figures 6–4). In November 2020, the PMMoV RNA load was 9.71 × 10^3^ ± 5.67 × 10^3^ copies/L. January 2021 increased to 1.16 × 10^4^ ± 2.50 × 10^3^ copies/L. February 2021 exhibited a similar level at 9.62 × 10^3^ ± 5.40 × 10^3^ copies/L. March 2021 recorded a substantial increase to 9.86 × 10^4^ ± 1.99 × 10^5^ copies/L. April 2021 displayed a decrease to 2.66 × 10^3^ ± 1.31 × 10^3^ copies/L. In May 2021, the RNA load was 9.07 × 10^3^ copies/L.

### Sororities

The RNA load of SARS-CoV-2 in 14 sororities displayed varying levels from November 2020 to May 2021 (Figure 5). In November 2020, the RNA load was 3.46 × 10^2^ ± 3.18× 10^2^ copies per liter (copies/L). January 2021 decreased to 7.34 × 10^1^ ± 7.55 × 10^1^ copies/L, indicating a substantial reduction. February 2021 recorded a further decrease to 4.41× 10^1^ ± 2.78× 10^1^ copies/L. March 2021 exhibited a modest increase to 2.02 × 10^2^ ± 2.15× 10^2^ copies/L. April 2021 displayed a lower RNA load at 2.09 × 10^1^ ± 1.79× 10^0^ copies/L. In May 2021, the RNA load was non-detectable, signifying a significant decrease or absence of viral presence in the raw sewage samples from sororities during that specific month.

Concurrently, the RNA load of PMMoV in sororities displayed varying levels from November 2020 to May 2021 (Figure 5). In November 2020, the PMMoV RNA load was 3.84 × 10^3^ ± 3.09 × 10^3^ copies/L. January 2021 increased to 6.97 × 10^3^ ± 3.81 × 10^3^ copies/L. February 2021 exhibited a similar level at 6.64 × 10^3^ ± 1.35 × 10^3^ copies/L. March 2021 decreased to 5.18 × 10^3^ ± 1.70 × 10^3^ copies/L. April 2021 displayed a further decrease to 4.86 × 10^3^ ± 4.53 × 10^3^ copies/L. In May 2021, the RNA load was 2.60 × 10^3^ copies/L.

### Impacts of normalization on correlations between SARS-CoV-2 RNA load and COVID-19 cases

Tables 1, 2 provide a comprehensive summary of the Spearman correlation (*r_s_*) values, elucidating the strength of associations between unnormalized and normalized SARS-CoV-2 RNA load by PMMoV and various factors such as active cases, self-isolations, and the combination of active cases with self-isolations. The analysis encompasses all residence halls, dormitories, fraternities, and sororities. Interpretation of *r_s_* values adheres to the criteria outlined by von Sperling et al. (2020). Correlations with a *r_s_* exceeding 0.7 are considered strong; those ranging between 0.4 and 0.7 indicate moderate correlations and values falling between 0 and 0.4 denote weak associations. Negative values signify weakened associations, while positive values suggest improved associations.

**Table 2:** Spearman correlation coefficients (*r_s_*) and significance *p*-values of wastewater measures (one-week prior average, at the week, and one-week after) against active, self-isolation and active cases plus self-isolation data without normalization.^a^

		Week prior		Week off		Week after	
		*r_s_*	*P -value*	*r_s_*	*P*-value	*r_s_*	*P*-value
Active cases	All	0.389	0.018*	0.472	0.004**	0.438	0.008**
	Dorms	0.399	0.017*	0.447	0.006**	0.398	0.018*
	Fraternities	0.428	0.023*	0.478	0.009**	0.478	0.009**
	Sororities	0.238	−	0.321	−	0.321	−
Self-isolations	All	0.286	−	0.313	−	0.263	−
	Dorms	0.194	−	0.257	−	0.192	−
	Fraternities	0.398	0.036*	0.450	0.014*	0.450	0.014*
	Sororities	0.414	0.036*	0.477	0.012*	0.477	0.012*
Active cases + Self-isolations	All	0.324	−	0.371	0.026*	0.323	−
	Dorms	0.268	−	0.325	−	0.267	−
	Fraternities	0.424	0.025*	0.473	0.010*	0.473	0.010*
	Sororities	0.375	−	0.443	0.021*	0.443	0.021*

^a^Significant correlations reflected as *p < 0.05 and **p < 0.01, and “−” reflects nonsignificant correlation.

Active cases, both independently and in combination with self-isolations, demonstrated noteworthy correlations with SARS-CoV-2 RNA load across all residential halls, spanning from moderate to weak levels (*r_s_* = 0.472, *p* = 0.004; *r_s_* = 0.313, *p* = 0.063; *r_s_* = 0.371, *p* = 0.026). Post-normalization, the correlations involving either active cases alone or the combined metric of active cases with self-isolations and SARS-CoV-2 RNA load diminished, revealing no statistically significant associations.

In dormitories, active cases displayed significant correlations with SARS-CoV-2 RNA load, characterized by moderate levels (*r_s_* = 0.447, *p* = 0.006). Nevertheless, these correlations waned after normalization by PMMoV, resulting in statistically insignificant associations with SARS-CoV-2 RNA load.

Within fraternities, active cases, self-isolations, and their combination exhibited substantial correlations with SARS-CoV-2 RNA load, marked by moderate correlation levels (*r_s_* = 0.478, *p* = 0.009; *r_s_* = 0.450, *p* = 0.014; *r_s_* = 0.473, *p* = 0.010). However, normalization by PMMoV led to a reduction in these correlations, rendering the associations with SARS-CoV-2 RNA load statistically insignificant.

Similarly, self-isolations and the combined metric of active cases with self-isolations showed significant correlations with SARS-CoV-2 RNA load within sororities, characterized by moderate correlation levels (*r_s_* = 0.321, *p* = 0.103; *r_s_* = 0.477, *p* = 0.012; *r_s_* = 0.443, *p* = 0.021). Yet, normalization by PMMoV resulted in a reduction of these correlations, leading to statistically insignificant associations with SARS-CoV-2 RNA load.

### Impacts of normalization correlations and early warning of COVID-19 cases trend

Tables 1, 2 elucidate the differences in *r_s_* values between unnormalized and normalized datasets. These values encapsulate the correlations between SARS-CoV-2 RNA load and various factors including active cases, self-isolations, and the combination of active cases with self-isolations. The analysis includes both a seven-day advance and a seven-day lag to discern lead times between WBE data and clinical cases, specifically emphasizing diverse student residence halls.

Preceding SARS-CoV-2 RNA load by one week demonstrated weakly significant correlations with active cases across all residential halls, while one week after, moderate correlations were observed with active cases (*r_s_* = 0.398, *p* = 0.018; *r_s_* = 0.438, *p* = 0.008). Importantly, weakly insignificant correlations were noted between the one-week advance and one-week lag of SARS-CoV-2 RNA load and both self-isolations and the combination with active cases.

Similarly, a seven-day advance and a seven-day lag of SARS-CoV-2 RNA load revealed weakly significant correlations with active cases across dormitories (*r_s_* = 0.399, *p* = 0.017; *r_s_* = 0.398, *p* = 0.018). Notably, weakly insignificant correlations were observed between the one-week advance and one-week lag of SARS-CoV-2 RNA load and both self-isolations and the combination with active cases (Table 3).

**Table 3:** Spearman correlation coefficients (*r_s_*) and significance *p*-values of wastewater measures (one-week prior average, at the week, and one week after) against active, self-isolation, and active cases plus self-isolation data with normalization (SARS-CoV-2/PMMoV).^a^

		Week prior		Week off		Week after	
		*r_s_*	*P*-value	*r_s_*	*P*-value	*r_s_*	*P*-value
Active cases	All	0.123	−	0.119	−	0.213	−
	Dorms	−0.223	−	−0.175	−	0.060	−
	Fraternities	0.021	−	0.178	−	0.168	−
	Sororities	0.246	−	0.269	−	0.157	−
Self-isolations	All	0.286	−	0.009	−	−0.010	−
	Dorms	−0.251	−	−0.395	0.046*	−0.391	−
	Fraternities	0.036	−	−0.137	−	−0.049	−
	Sororities	0.398	−	0.216	−	0.077	−
Active cases + Self-isolations	All	0.272	−	0.041	−	0.068	−
	Dorms	−0.262	−	−0.326	−	−0.253	−
	Fraternities	0.051	−	−0.081	−	0.000	−
	Sororities	0.402	−	0.225	−	0.112	−

*Significant *P*-value.

A seven-day advance and a seven-day lag of SARS-CoV-2 RNA load exhibited moderately significant correlations with active cases and the combination with self-isolations among fraternities (*r_s_* = 0.418, *p* = 0.023; *r_s_* = 0.478, *p* = 0.009; *r_s_* = 0.424, *p* = 0.025; *r_s_* = 0.473, *p* = 0.010). Furthermore, weakly significant correlations were identified between the one-week advance of SARS-CoV-2 RNA load and self-isolations, while a moderate correlation was observed for the one-week lag.

A seven-day advance and a seven-day lag of SARS-CoV-2 RNA load exhibited moderately significant correlations with self-isolations among sororities (*r_s_* = 0.414, *p* = 0.036; *r_s_* = 0.477, *p* = 0.012). Furthermore, moderate significant correlations were identified between the one-week lag of SARS-CoV-2 RNA load and the combination of active cases and self-isolations.

Normalization by PMMoV resulted in either a reduction or an insignificant impact on *r_s_* values for a seven-day advance and a seven-day lag of SARS-CoV-2 RNA load with active cases, self-isolations, and their combinations across all building types in this study.

## Discussion

Integrating WBE into public health surveillance is progressing as a strategic response to the challenges posed by the COVID-19 pandemic. A crucial aspect of this approach is understanding the correlations between SARS-CoV-2 RNA load and COVID-19 cases, particularly in communities characterized by mobile populations and significant temporal fluctuations. This significance is accentuated in situations where reported clinical case numbers may not accurately reflect the actual residents in these communities.

In our study, we observed notable variations in SARS-CoV-2 RNA load among different building types, irrespective of whether they accommodated high (>200) or low (<50) student numbers in the corresponding residence halls. Our findings underscore a consistent alignment between SARS-CoV-2 RNA load in raw sewage from all student residence halls, as assessed through RT-qPCR, and the trends observed in active cases of COVID-19 across all residence halls. These results are in harmony with existing research, affirming that SARS-CoV-2 RNA load serve as an effective tool for estimating the dynamics of COVID-19 cases within diverse communities. Medema et al. (2020) reported a significant correlation between SARS-CoV-2 RNA load and cumulative COVID-19 prevalence, even in situations of low prevalence in the Netherlands. Xiao et al. (2022) documented that SARS-CoV-2 RNA load preceded new cases during the first wave of COVID-19 in MA, USA. Additionally, Ai et al. (2021) found a robust correlation between SARS-CoV-2 RNA load and confirmed cases in 8 sewersheds. Wurtzer et al. (2020) similarly reported correlations between SARS-CoV-2 RNA load and the number of symptomatic or non-symptomatic COVID-19 patients at local or regional scales. Furthermore, Schmitz et al. (2021) discovered a correlation between clinical data and corresponding sewage SARS-CoV-2 RNA load in student dorms at the University of Arizona during the fall semester of 2020.

Given that dormitories, fraternities, and sororities represented as microcosms of the campus, our study revealed that the SARS-CoV-2 RNA load identified in dormitories and fraternities correspondingly mirrored the trends observed in COVID-19 cases across all student residence halls. The greater proportion of undergraduates residing in dormitories might provide a more complete picture of the COVID-19 situation on campus. Fraternities may be factored into the analysis due to the correlation between males and the presence of fecal viruses (Daou et al., 2022). This correlation is consistent with the research conducted by Bitter et al. (2022), wherein the university was identified as a microcosm of the corresponding city and exhibited analogous trends in COVID-19 cases. Furthermore, our analysis revealed that Spearman rank correlations ranged from 0.447 to 0.478, with no relation to the size of the student residence halls, consistent with the findings of Feng et al. (2021), who noted similar correlations unrelated to the size of wastewater treatment plants. While our findings consistently showed alignment between SARS-CoV-2 RNA load in raw sewage from all student residence halls, as determined through RT-qPCR, and the trends observed in active cases of COVID-19 across these halls, our study relied solely on RNA load as a proxy for COVID-19 prevalence, which may not capture all cases, particularly those of asymptomatic individuals or those with low viral shedding. Therefore, caution should be exercised when interpreting and extrapolating our results to other settings or populations.

While the normalization of SARS-CoV-2 RNA load in wastewater by PMMoV has the potential to enhance comparability across studies, our investigation demonstrated that there was no substantial improvement in correlations between SARS-CoV-2 RNA load in raw wastewater and active cases, self-isolation, or their combination when compared to unnormalized data, irrespective of the student population in the respective residence halls. The results of this study add to the expanding body of research that questions the notion that normalization by PMMoV enhances the reporting and standardization of WBE data. Ai et al. (2021) reported that normalization by PMMoV did not significantly improve correlations with new case numbers or enhance estimation models across nine wastewater treatment plants serving populations ranging from 14,000 to 900,000. Similarly, Feng et al. (2021) found that normalizing SARS-CoV-2 RNA load to PMMoV did not improve correlation coefficients in all 12 WWTPs serving populations from 11,000 to 616,000. In the study by Greenwald et al. (2021), normalization by PMMoV led to a reduction in significant correlations to clinical cases in wastewater catchment areas serving populations from 2,930 to 1,500,000. A recent study by Nagarkar et al. (2022) found that PMMoV had a stronger correlation with samples from a larger sewershed, which served approximately 488,000 people and had higher levels of industrial and stormwater inputs. Zambrana et al. (2022) found that normalizing SARS-CoV-2 RNA load by PMMoV did not substantially change the correlation coefficients at various levels, including campus level (serving around 10,000 people), building level (serving around 500 people), or individual-building level (serving around 200 people) within the Stanford University campus. These findings contrast with other studies suggesting that normalization to PMMoV enhances relationships with clinical data. Other than PMMoV, Hsu et al. (2022) found that Paraxanthine was a more reliable population biomarker than PMMoV. Mitranescu et al. (2022) reported that the best normalization performance was achieved with a mixed fecal indicator calculated from both CrAssphage virus and PMMoV. It is crucial to note that these divergent conclusions were primarily attributed to the choice of matrix (solids versus liquid) (D'Aoust et al., 2021; Wolfe et al., 2021) and variances in analytical methods (Scott et al., 2021; Zhan et al., 2022).

Furthermore, the choice of molecular method/kit, particularly quantitative PCR, employed for the detection of SARS-CoV-2 and/or PMMoV RNA, may also influence the correlations between the two viruses. For instance, Jafferali et al. (2021) conducted a study comparing four concentration methods, including two variants of ultrafiltration-based methods and two adsorption and extraction-based methods. They consistently observed higher recovery of SARS-CoV-2 and PMMoV using the modified ultrafiltration-based method. In our study, we utilized the ultracentrifugation method, which, while effective, may be slightly less efficient than the modified method. Moreover, the choice of target genes (N1, N2, and E) for detecting SARS-CoV-2 can impact the performance of the assay, with different extraction methods yielding varying recoveries for these genes. Nalla Arun et al. (2020) conducted a comparative analysis of SARS-CoV-2 detection assays using seven different primer-probe sets and one assay kit. They found that the most sensitive assays utilized the E-gene primer-probe set described by Corman et al. (2020) and the N2 set developed by the CDC. In our study, we opted for the N1 target due to consistent contamination issues with the N2 set in our laboratory. More sensitive kits and/or advanced methods should be considered for future studies to enhance the accuracy and reliability of such comparisons.

Environmental uncertainties introduce complexities in normalizing SARS-CoV-2 RNA load in raw wastewater by PMMoV. Raw wastewater from university dormitories primarily consists of washing and bathing effluents, occasionally mixed with kitchen wastewater. In contrast, sewage wastewater derives from a broader spectrum, including residential sources (private residences, dormitories, hotels, and residential care facilities) and commercial facilities (including hospitals), creating a more intricate composition compared to university dormitory wastewater. Hamouda et al. (2021) underscored the role of microorganisms and physico-chemical properties, such as pH, solids, and disinfectants, in influencing the persistence and detection of SARS-CoV-2 RNA in wastewater. These factors impact the genetic material’s integrity, posing challenges in detection. Higher pH in wastewater has been linked to decreased RNA load of all three SARS-CoV-2 targets (Ahmed et al., 2020b; Maal-Bared et al., 2023). Sapula et al. (2021) reported the non-detection of SARS-CoV-2 in a wastewater plant with elevated pH levels (8.80 to 9.35). These findings also have implications for wastewater treatment plants receiving waste with high pH (pH > 7.75) from various sources, including lagoons, septic tanks, and industrial operations. Elevated pH levels can significantly affect virus adsorption to particles and the RNA load recovered, emphasizing the need for careful consideration of wastewater composition and pH variations in interpreting surveillance data. Accurate interpretation of WBE relies on addressing environmental uncertainties in both raw wastewater and influent wastewater.

The correlation between SARS-CoV-2 RNA load and PMMoV normalization may be influenced by the distinct characteristics of the two viruses in terms of their source and origin. SARS-CoV-2 is an enveloped virus, while PMMoV is non-enveloped. In contrast to wastewater transported to treatment plants, our study collected wastewater directly from manholes next to the buildings or from access points before the sewer pipe left the building and promptly processed it in the lab. This immediate processing could affect virus RNA load due to variations in decay rates and variability between the enveloped SARS-CoV-2 and non-enveloped PMMoV. Research conducted by Li et al. (2023b) has indicated a faster decay rate of SARS-CoV-2 RNA compared to PMMoV RNA in raw sewage. PMMoV RNA exhibited higher abundance and lower variability than pathogenic SARS-CoV-2 RNA (Li et al., 2023b). A study by Ye et al. (2016) found that up to 26% of enveloped viruses (murine hepatitis virus and *Pseudomonas* phage φ6) adsorbed to the solid fraction of wastewater, compared to 6% of non-enveloped viruses (Bacteriophage MS2 and T3). This implies that enveloped viruses may maintain greater integrity in wastewater with higher total suspended solids (TSS) and turbidity levels. Recognizing the distinct characteristics of enveloped and non-enveloped viruses is crucial for enhancing the precision and meaningfulness of interpretation the data in the context of WBE studies.

The study investigated the impact of normalizing SARS-CoV-2 RNA load by PMMoV on lead times associated with active cases, self-isolation, or their combination. The significant correlations observed one week prior when analyzing SARS-CoV-2 RNA load from all residence halls in this study, indicating a potential early warning utility. These results suggest that SARS-CoV-2 RNA load may serve as an early indicator, aligning with findings from other studies. Nemudryi et al. (2020) reported that SARS-CoV-2 RNA load in wastewater precedes clinical test results by 2 to 4 days at the community level. Greenwald et al. (2021) found that the strongest correlation between clinical testing data and SARS-CoV-2 RNA load was associated with a two-week lead time across six wastewater catchment areas. Karthikeyan et al. (2021) observed a strong correlation between SARS-CoV-2 RNA load in raw untreated wastewater and clinically cases at the county level. The integration of this correlation, along with historical reported case numbers and temporal information in an autoregressive integrated moving average model, facilitated the successful prediction of new reported cases up to 3 weeks in advance. Wu et al. (2022) found that SARS-CoV-2 RNA load in wastewater were correlated with clinically diagnosed new COVID-19 cases, revealing trends four to ten days earlier in wastewater than in clinical data during the initial wave of the pandemic at the Deer Island Wastewater Treatment Plant. However, Xiao et al. (2022) observed that this leading indicator effect was not apparent in the second wave from the same treatment plant. The authors attributed this change to the expansion of testing capacity, enabling more timely identification, and reporting of cases. Additionally, the diverse approaches to reporting cases, considering factors like disease onset, test date, and test result date, also contribute to the variability observed in lead times. Ai et al. (2021) emphasized this by noting that their clinical cases staggered the trend of SARS-CoV-2 RNA load by 3 days since positive cases were assigned a date based on the estimate of disease onset.

In the present study, normalizing SARS-CoV-2 RNA load by PMMoV did not result in enhancements of lead times associated with active cases, self-isolation, or their combination across all examined locations. Our observations are in alignment with the findings of Maal-Bared et al. (2023), who reported that the normalization of SARS-CoV-2 RNA load by PMMoV did not improve lead times for active cases in ten out of twelve communities.

The correlations observed were significantly weaker between the SARS-CoV-2 RNA load one week before and the occurrence of active cases, self-isolation, or their combination in all student residence halls. The strongest correlations were observed for comparisons with the SARS-CoV-2 RNA load during the week of and one week after the occurrence of active cases in all student residence halls. Consistent with our findings, Ai et al. (2021) also reported no enhancement in correlations for the estimation of leading times. This lack of improvement may be attributed to viral load variations associated with disease progression (Benefield et al., 2020; Walsh et al., 2020). Zheng et al. (2020) further emphasized that stool sample viral loads were highest during the third and fourth weeks after disease onset.

While our study provides valuable insights into the limitations of normalizing SARS-CoV-2 RNA load by PMMoV in wastewater, it is essential to acknowledge several limitations in our methodology and data interpretation. Firstly, the study was conducted within a specific context of university dormitories, which may not fully represent the broader community dynamics of wastewater surveillance. The findings may not be directly generalizable to other settings with different population densities, wastewater characteristics, and public health interventions. Additionally, the observational nature of the study limits our ability to establish causality between RNA load measurements and epidemiological outcomes. Other factors such as testing strategies, reporting practices, and population mobility could also influence the observed correlations and lead times. Furthermore, the study focused primarily on correlations between RNA load and clinical outcomes without considering other potential confounders or modifiers that could affect the relationship. Future research incorporating more comprehensive data sources and analytical methods could provide a more nuanced understanding of the utility of PMMoV normalization in predicting COVID-19 dynamics. Lastly, while our study contributes to the existing literature on wastewater surveillance, it is essential to recognize that WBE is just one component of a broader public health strategy and should be integrated with clinical data and epidemiological models for effective disease monitoring and control.

## Conclusion

This year-long study monitored SARS-CoV-2 RNA load in the raw sewage of a university campus, aiming to understand the correlations with various aspects, including active COVID-19 cases, self-isolations, and their combination among all residence students. The findings revealed significant positive correlations between SARS-CoV-2 RNA load a week prior and during the week of monitoring from all student residence halls, dormitories, and fraternities and the occurrence of active COVID-19 cases among all residence students. This suggests that raw sewage monitoring can be a valuable tool for early warning and outbreak tracking within the campus setting. However, normalizing data using the fecal marker PMMoV did not demonstrate clear utility in interpreting campus-wide data. These results contribute to our understanding of the potential applications and limitations of wastewater monitoring for COVID-19 surveillance within the context of raw sewage on university campuses.

## Data availability statement

The raw data supporting the conclusions of this article will be made available by the authors, without undue reservation.

## Author contributions

YL: Conceptualization, Data curation, Formal analysis, Investigation, Methodology, Validation, Visualization, Writing – original draft, Writing – review & editing. KA: Data curation, Formal analysis, Investigation, Methodology, Software, Visualization, Writing – review & editing. IA: Formal analysis, Investigation, Methodology, Visualization, Writing – review & editing. DJ: Funding acquisition, Investigation, Methodology, Project administration, Resources, Software, Supervision, Writing – review & editing. DW: Investigation, Methodology, Resources, Validation, Writing – review & editing. PM: Data curation, Investigation, Methodology, Resources, Writing – review & editing. BG: Formal analysis, Investigation, Methodology, Resources, Writing – review & editing. SD: Formal analysis, Investigation, Methodology, Validation, Writing – review & editing. CN: Data curation, Formal analysis, Investigation, Methodology, Resources, Software, Validation, Visualization, Writing – review & editing. FK-M: Investigation, Methodology, Resources, Writing – review & editing. CS: Investigation, Methodology, Resources, Writing – review & editing. TH: Conceptualization, Data curation, Formal analysis, Funding acquisition, Investigation, Methodology, Project administration, Resources, Software, Supervision, Validation, Visualization, Writing – original draft, Writing – review & editing.
